# Drug resistance patterns in new and previously treated patients with tuberculosis presenting to a tertiary care center in southern India

**DOI:** 10.1186/1471-2334-14-S3-O3

**Published:** 2014-05-27

**Authors:** Anto J Udaykumar, Jayashree R, Baijayanti Mishra, Rosario Vivek, John Kenneth

**Affiliations:** 1 Division of Infectious Diseases, St. John’s Research Institute, Bangalore 560034, India; 2 Department of Microbiology, St. John’s Medical College Bangalore 560034, India

## Background

Tuberculosis (TB) caused by *Mycobacterium tuberculosis* remains a major global health problem. It is the second leading cause of death worldwide. Drug resistance threatens global TB control with the emergence of multidrug resistant (MDR) and extensively drug resistant (XDR) TB. The burden of MDR TB and XDR TB in India is not available as continuous surveillance for drug resistance is not carried out. This study was done to determine the drug resistance patterns to first and second line drugs among new and previously treated patients with TB.

## Methods

Sputum samples were collected from 100 suspected TB patients from a tertiary care referral hospital. After digestion and decontamination, samples were stained for microscopy and cultured in liquid (MGIT) and Löwenstein Jensen (LJ) medium to isolate *M. tuberculosis*. Drug susceptibility testing (DST) was done against first line (streptomycin, isoniazid, rifampin and ethambutol) and second line (kanamycin, capreomycin, ofloxacin and ethionamide) drugs.

## Results

Among the 100 samples, 56 were smear positives and *M. tuberculosis* was isolated from 61 samples cultured in MGIT and 46 in L J medium. Of which 20 (32.7%) were MDR strains. Among the MDR strains, 9 (45%) were XDR strains. Two strains were totally resistant to all the 8 drugs. The proportion of MDR and XDR was highest among the previously treated patient.

## Conclusion

This study underscores the need for DST in all TB patients particularly in the previously treated patients. New drugs, novel treatment strategies and adherence to treatment are needed to effectively treat and control drug resistant TB.

